# Oral microbiota and biliary tract cancers: unveiling hidden mechanistic links

**DOI:** 10.3389/fonc.2025.1585923

**Published:** 2025-06-11

**Authors:** Yuhan Zhang, Shu Zhang

**Affiliations:** ^1^ Nursing Department, The First Affiliated Hospital of Chongqing Medical University, Chongqing, China; ^2^ College of Stomatology, Chongqing Medical University, Chongqing, China

**Keywords:** oral microbiota, oncogenic bacteria, biliary tract cancer, bacterial translocation, mechanisms

## Abstract

Biliary tract cancers (BTCs), a group of rare aggressive malignancies, posed significant clinical challenges due to late diagnosis and limited therapies. While gut microbiota had been extensively studied in gastrointestinal cancers, the role of oral microbiota—a primary microbial reservoir entering the digestive system—remained poorly understood. Emerging evidence indicated that oral bacteria might affect biliary carcinogenesis through direct colonization, immune modulation, and metabolic interactions via the oral-gut-liver axis. This narrative review analyzed current research connecting oral microbial imbalance with BTCs. It explored how bacterial translocation, inflammatory metabolites, and immune alterations could promote cancer development. Established BTC risk factors—including gallstones, primary sclerosing cholangitis, cirrhosis, and *H. pylori* infection—were evaluated for their associations with oral microbiota changes. Epidemiological studies revealed that periodontal disease and poor oral hygiene elevated BTC risk. Sequencing analyses identified oral-origin bacteria (*Prevotella*, *Fusobacterium*, *Streptococcus*) in bile and tumor tissues, suggesting microbial migration through swallowing or bloodstream. Mechanistic investigations showed microbial components (e.g., lipopolysaccharides, membrane vesicles) activated inflammatory pathways (TLR4/NF-κB, STAT3) and modified immune checkpoints, while metabolites potentially altered biliary cell metabolism. Different studies have found variable changes in oral microbiota in the presence of BTCs, thus a novel “biphasic dysbiosis” hypothesis was proposed to explain differing oral microbial diversity patterns across BTC subtypes. Despite progress, critical knowledge gaps persisted regarding causality, spatial microbial variations, and functional impacts of metabolites in BTCs. Future research was recommended to employ multi-omics approaches, single-cell analysis, and AI tools to enhance early detection and prevention strategies.

## 1 Introduction

The human microbiota encompassed diverse communities of microorganisms that exist in association with human hosts, comprising microbes from various domains of life (1). These microbial populations, spanning bacteria, fungi, viruses, and archaea, play a dual role in host biology. On one hand, it played essential roles in maintaining host homeostasis through immune modulation, nutrient metabolism, and epithelial barrier integrity. On the other, dysbiosis—an imbalance in microbial composition—has been implicated in oncogenesis via chronic inflammation, metabolic reprogramming, and immune evasion (2). Microbiota composition is highly site-specific, varying considerably across different anatomical locations such as the skin, oral cavity, gut, and biliary tract. Each niche harbors a unique microbial ecosystem influenced by age, environment, diet, genetics, and disease states. The interplay between microbiota and host immunity represents a critical determinant of health status, with alterations in local microbial compositions being extensively associated with various pathological conditions (3). Of these, the oral cavity is of particular interest as it constitutes the second largest microbial habitat in the body and serves as the initial gateway to the gastrointestinal system. The oral microbiota comprises more than 700 bacterial species, alongside fungi and viruses, and its balance can be disrupted by factors such as tobacco use, alcohol consumption, poor dental hygiene, periodontitis, and prolonged antibiotic exposure (4).

Biliary tract cancers (BTCs) represented a heterogeneous group of malignant neoplasms originating from the epithelial cells of the biliary system, including intrahepatic cholangiocarcinoma (iCCA), perihilar cholangiocarcinoma (pCCA), distal cholangiocarcinoma (dCCA) and gallbladder cancer. Despite their relatively low overall incidence in the general population, BTCs demonstrate an increasing global trend, with notably higher prevalence in Asian regions (5). These malignancies presented significant clinical challenges due to their asymptomatic early stages, frequent late-stage diagnosis post-metastasis, high chemotherapy resistance, and limited targeted therapeutic options. As a result, their five-year survival rate remains below 30%, underscoring the urgent need for improved prevention and early diagnostic strategies (6, 7).

The tumor microenvironment constitutes a complex ecosystem comprising both host and microbial cells associated with neoplastic tissue, in which resident microbiota actively participated in modulating cancer progression. Emerging evidence illustrated how microbiota shaped the tumor microenvironment through direct contact with host tissues or via secreted metabolites (8, 9). For example, butyrate, a short-chain fatty acid produced by bacterial fermentation of dietary fiber, demonstrated anti-tumor properties. It promoted cancer cell apoptosis and inhibited tumor growth. These effects were mediated through multiple mechanisms, including histone deacetylase (HDAC)—the enzymes that control gene expression through chromatin remodeling—inhibition, G protein-coupled receptor activation, and cellular metabolism regulation (10).

While most microbiome-oncology studies have centered on the gut, growing evidence suggested that oral microbes might also influence systemic disease processes. With evidence has established that various pathophysiological factors can induce oral microbial dysbiosis, which include tobacco use, alcohol consumption, prolonged antibiotic administration, dental caries, and periodontitis. Such disruptions to the oral microbiome have significant implications for oral health (11). As the initial segment of the digestive tract, oral microbiota can translocate to downstream digestive organs via swallowing (the oral-gut axis), entering systemic circulation through inflamed or damaged oral tissues (the oral-blood axis) or other ways (12, 13). These translocated microbes and their metabolites could affect distant organs, including the liver and biliary system, by disrupting local microbial communities, triggering inflammatory responses, or altering metabolic pathways. Recent investigations have increasingly demonstrated both direct and indirect associations between oral microbiota (particularly *Porphyromonas gingivalis* and *Fusobacterium nucleatum*) and various digestive system disorders, including colorectal cancer, inflammatory bowel disease, and chronic liver diseases (14–17).

While significant advances have been made in understanding the relationship between microbiota and digestive system malignancies, the potential connection between oral microbiota and BTCs-a significant component of digestive system cancers-remained incompletely elucidated. Understanding this connection could open new avenues for early diagnosis and intervention, particularly given the oral cavity’s accessibility for non-invasive sampling.

This narrative review aimed to comprehensively examine the mechanistic and clinical associations between the oral microbiota and BTCs. We focused on the translocation pathways, metabolic and immunological interactions, and the impact of oral microbial dysbiosis on BTC risk and progression. We further evaluated the relationship between established BTC risk factors—such as cholelithiasis, primary sclerosing cholangitis (PSC), liver cirrhosis, and Helicobacter pylori infection—and oral microbial alterations. Our review synthesized findings from observational studies, microbiome sequencing analyses, and mechanistic investigations. The study was conducted with comprehensive searches performed in PubMed and Web of Science databases from 2000 to April 17, 2025. The search terms included: (“oral microbiota” OR “oral microbiome” OR “oral flora” OR “oral bacteria”) AND (“biliary tract cancer” OR “biliary tract neoplasm” OR “cholangiocarcinoma” OR “bile duct cancer” OR “gallbladder cancer”). Additional searches were conducted using terms related to established risk factors: (“oral microbiota” OR “oral microbiome” OR “oral flora” OR “oral bacteria”) AND (“cholelithiasis” OR “primary sclerosing cholangitis” OR “liver cirrhosis” OR “Helicobacter pylori”).

By consolidating and critically interpreting the current evidence, this review aims to clarify the biological relevance of the oral microbiota in BTC development and highlight future research directions. In doing so, it seeks to make this emerging field more accessible to interdisciplinary audiences spanning oncology, microbiology, hepatology, and oral medicine.

## 2 Normal oral microbiota

The oral cavity represents a complex microbial ecosystem, serving as the second largest microbial reservoir in the human body. Advanced DNA analysis of the oral microbiome has revealed the presence of over 700 distinct bacterial species. Oral microbial genera and species vary between individuals due to environmental factors. However, the predominant bacterial phyla remain relatively consistent in healthy individuals. This conservation of main microbiota composition in health states provided valuable insights into understanding the relationship between oral and systemic health (11, 18).

The microbiota comprises various microorganisms, including bacteria, fungi, viruses, archaea, and prokaryotes, but current research predominantly focused on bacterial communities. Contemporary technological advances, including 16S rRNA high-throughput sequencing, metagenomics, single-cell genomics, and integrated multi-omics analysis, enabled comprehensive and precise characterization of oral microbiota structure and function. Current evidence established the major bacterial phyla in the oral cavity, in order of prevalence: *Firmicutes* (including *Streptococcus*, *Gemella*, *Eubacterium*, *Selenomonas*, *Veillonella*), *Actinobacteria* (including *Actinomyces*, *Atopobium*, *Rothia*), *Proteobacteria* (including *Neisseria*, *Eikenella*, *Campylobacter*), *Bacteroidetes* (including *Porphyromonas*, *Prevotella*, *Capnocytophaga*), *Fusobacteria* (including *Fusobacterium* and *Leptotrichia*), *TM7*, *Spirochaetes*, *OD2*, and *Synergistetes*. The predominant genera include *Streptococcus*, *Haemophilus*, *Neisseria*, *Prevotella*, *Veillonella*, and *Rothia* (19, 20).

## 3 Oral microbiota and BTCs

**Figure 1:**
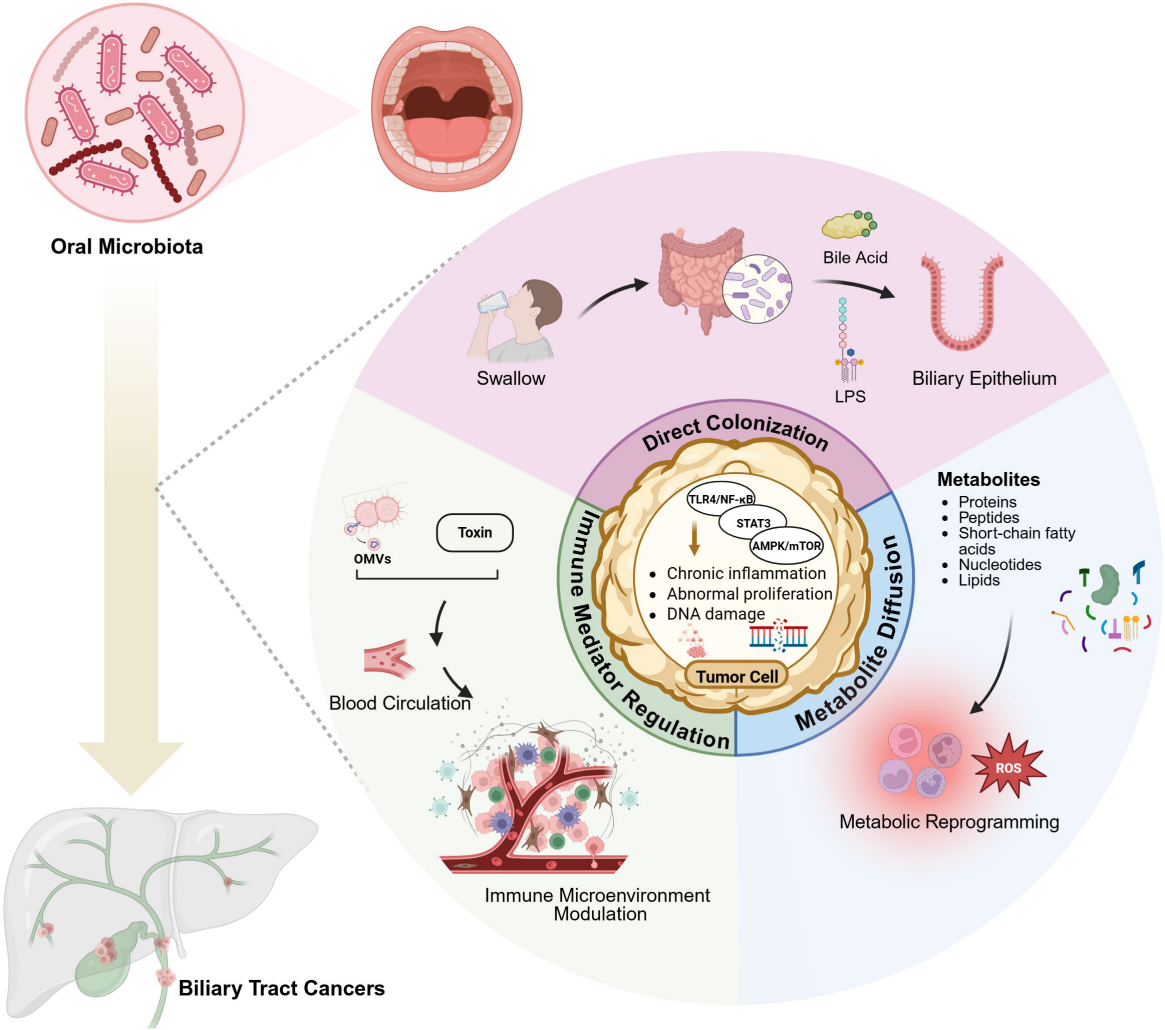
Possible oral microbiota-mediated pathways in biliary tract cancer development. Through the oral-gut axis, oral microorganisms directly colonize intestinal mucosa via swallowing. Their metabolites, including secondary bile acids and LPS, influence the hepatobiliary system through the gut-liver axis. Microbial metabolites interact with host cell receptors to modulate local inflammatory responses and cellular metabolism through metabolic reprogramming. Bacterial genera such as Streptococcus and Prevotella survive in the bloodstream, where their secreted toxins and bacterial outer membrane vesicles (OMVs) modulate host immune responses and reshape the cancer immune microenvironment. Created in https://BioRender.com.

The oral cavity, serving as the initial segment of the digestive tract, functioned as an endogenous reservoir for gut microbiota. Oral microorganisms demonstrated multiple pathways of translocation to the digestive tract through direct colonization, immune mediator regulation, and metabolite diffusion. Through the oral-gut axis, oral microbiota directly migrated and colonized the intestinal mucosa via swallowing and blood circulation (21). Upon entering the intestinal tract, oral microbiota interact with resident gut microbes and alter the intestinal microbial composition and immune microenvironment through mechanisms such as competition, symbiosis, and nutrient sharing (21, 22). Once established in the gut, oral microbiota can modulate the enterohepatic circulation of bile acids, influencing their conversion into secondary bile acids by the intestinal microbiota. These secondary bile acids affect liver metabolism and immune responses, leading to altered bile acid signaling through FXR and TGR5, which may contribute to chronic inflammation and immune dysregulation, potentially promoting the development of BTCs (23). Moreover, gut microbiota and their metabolites, including short-chain fatty acids, secondary bile acids, and lipopolysaccharides (LPS), exert significant effects on the hepatobiliary system through gut-liver axis signaling mechanism (24). (See **Figure 1** for schematic representation of these pathways).

Oral microbiota exerted indirect influence on digestive organs through the production of diverse metabolites (e.g., proteins, peptides, short-chain fatty acids, nucleotides, lipids). Through interactions with host cell receptors, these metabolites can modulate local inflammatory responses, metabolic functions, and even carcinogenic processes. For instance, butyrate produced by *Prevotella* was believed to regulate intestinal immune barriers, while LPS produced by *Veillonella* could exacerbate chronic inflammatory conditions in the biliary tract (13).

Poor periodontal health is associated with alterations in oral microbial diversity (25). And in a prospective study of 65,869 women, a history of periodontal disease was significantly associated with an elevated risk of BTCs (hazard ratio, 1.73; 95% confidence interval, 1.01-2.95) (26). Additionally, a United Kingdom-based cohort study involving 286 BTCs cases revealed that self-reported poor oral health correlated with increased risk of biliary system malignancy in unadjusted analyses (1.32; 95% CI, 0.95-1.80) (27). These findings suggested a potential relevance between oral microbiota, as key determinants of oral health, and increased BTCs risk.

Bile, a light yellow fluid synthesized by hepatocytes, comprises bile acids (BA), cholesterol, phospholipids, and proteins, and undergoes transport from the liver and gallbladder to the intestine via bile ducts (28). The biliary microbiota predominantly consists of *Firmicutes*, *Bacteroidetes*, *Actinobacteria*, and *Proteobacteria phyla*, with additional phyla including *Verrucomicrobia*, *Chlamydiae*, *Acidobacteria*, *Planctomycetes*, *Cyanobacteria*, *Spirochaetes*, and *Fusobacteria* present in lower proportions (0.05-0.5%) (29). In a study of 14 patients with pCCA, increased abundance of oral-associated *Prevotella genera* was observed. Analysis of 9 patients with dCCA revealed elevated levels of *Streptococcus*, *Prevotella*, and *Actinomyces genera* (30). Through 16S rRNA sequencing analysis of biliary microbiota from 8 dCCA patients, Chen et al. demonstrated that while overall phylum-level composition remained relatively stable, significant increases occurred in *Gemmatimonadetes*, *Nitrospirae*, and *Planctomycetes phyla* abundance, accompanied by notable reduction in *Chloroflexi* (31). In another 16S rRNA sequencing analysis involving 60 BTCs patients, *Dietzia* and *Pseudomonas* genera were identified as the predominant inhabitants of the biliary tract tumor tissue. Additionally, enrichment of *Bifidobacteriaceae* was observed in cases with Opisthorchis viverrini infection (32). Notably, *Dietzia* genera primarily constitute skin and oral microbiota components, while *Pseudomonas* and *Bifidobacteriaceae*, as opportunistic pathogens, may colonize the oral cavity through environmental exposure before subsequent biliary tract translocation (33, 34). In a multicenter case-control study encompassing 100 BTCs patients, Avilés-Jiménez et al. documented significant increases in *Fusobacterium* and *Prevotella* genera abundance, accompanied by decreased levels of *Rothia*, typically considered a normal oral microbiota constituent (35).

Uguz A et al. examined pancreatic samples from 10 patients with dCCA or ampullary cancer (AC). The microbiota was dominated by the phyla *Firmicutes*, *Actinobacteria*, *Proteobacteria*, *Bacteroidetes*, and *Acidobacteria*. *Firmicutes* were the most abundant, while *Bacteroidetes* and *Acidobacteria* were significantly enriched. At the genus level, microbial profiles varied markedly between individuals. Sequence alignments of saliva and pancreatic samples identified *Prevotella*, *Streptococcus*, and *Fusobacterium* as the oral genera most frequently enriched in pancreatic tissue (36). These findings further suggest the potential impact of oral microbiota on peritumoral BTC tissues.

Based on the gut-liver axis theory, intestinal microbiota and their metabolites can directly affect the hepatobiliary system through the portal venous circulation, participating in the development and progression of various diseases (37). In BTCs patients, increased abundances of *Veillonella*, *Parabacteroides*, and *Enterobacter* genera were observed, along with elevated levels of *Firmicutes* and *Actinobacteria* phyla, revealing the correlation between dysbiosis and carcinogenesis. Further studies demonstrated that the intestinal microbiota can drive BTCs progression through key regulatory points, including energy metabolism reprogramming and cell proliferation control via the AMPK and mTOR signaling pathways (38).

The mechanisms of oral bacterial metabolites in BTCs development require further investigation. For instance, *P. gingivalis*-derived LPS activates the TLR4/NF-κB pathway in microglial cells, triggering inflammatory cascades (39). And sustained inflammatory microenvironment promotes aberrant cell proliferation and carcinogenesis through multiple signaling pathways, including NF-κB and STAT3 (40). While it was relatively clear that the aforementioned oral microbiota metabolites might have influenced the development and progression of BTCs by modulating inflammatory pathways and metabolic reprogramming, their temporal distribution, precise concentrations, and specific targets within the biliary microenvironment had not been adequately clarified (41). With the advancement of relevant technologies, future studies urgently need to employ techniques such as isotope labeling-based metabolic analysis and targeted metabolomics analysis in both vivo and vitro experiments. These approaches are expected to clarify the local dynamic changes and specific mechanisms of these metabolites, thereby defining their precise roles in the pathogenesis of BTCs.

During BTCs development, changes occur not only in the biliary and gastrointestinal microbiota but also in the oral microbiome composition. A study of gallbladder cancer patients (n=272) demonstrated that these patients exhibited significantly higher α-diversity and abundance of rare species in their oral microbiota compared to healthy controls. They also successfully developed a high-reliability predictive model for BTCs’ probability using a marker set comprising three genera—*Actinomyces*, *Alloprevotella*, and *Lautropia*. This model revealed the feasibility of utilizing changes in the oral microbiota as an auxiliary diagnostic indicator for BTCs (42). However, another study of 10 patients with dCCA or ampullary cancer found that oral microbial diversity was lower in the cancer group than in healthy controls (36). Peculiarly, Oh S. et al. compared the oral microbiota of 14 BTC patients with 14 healthy controls and found no significant difference (p = 0.1) (43). Although variations in sample size, sequencing depth, and control of confounding factors may have caused these differences, Oh S. et al. observed that cancers at different anatomical sites produced distinct shifts in the oral microbiota. This finding demonstrated that the anatomical site determines the pattern of microbial change (43). It is acknowledged that iCCA is propelled by chronic inflammation and is characterized by a highly immunosuppressive tumor microenvironment, which includes elevated PD-L1 expression and MDSC accumulation (44). In this context, it is hypothesized that these mechanisms could weaken immune surveillance and can contribute to systemic immune dysregulation, potentially affecting sites such as the oral mucosa. Weakended immune surveillance allows rare oral bacteria taxa to flourish, increasing overall oral microbiota diversity. In contrast, dCCA is usually accompanied by bile duct obstruction and repeated biliary inflammation. These conditions often require early and frequent antimicrobial treatment, which suppresses some species and lets a few dominant pathogens take over, thereby reducing diversity (45, 46). In addition, factors such as malnutrition, oral dryness, reduced food intake, and preoperative antibiotic use in some advanced dCCA patients lowered oral microbial diversity. These findings also underscore the shortage of studies examining heterogeneity driven by anatomical subtypes. Future work should clarify the underlying pathways and involve large-scale, multicenter cohorts. (See **Table 1** for summary of clinical evidence regarding oral microbiota changes in BTC patients).

**Table 1:** Evidence of oral microbiota alterations in biliary tract cancer.

Authors	Year	Sample size	Key findings	Statistical data
Nwizu NN et al. (26)	2017	65,869 Women	History of periodontal disease associated with elevated risk of biliary tract cancer.	HR = 1.73 (95% CI: 1.01-2.95)
Jordão HW et al. (27)	2019	286 BTC cases	Self-reported poor oral health correlated with increased risk of biliary system malignancy.	HR = 1.32 (95% CI: 0.95-1.80)
Li Z et al. (30)	2022	14 pCCA cases	*Prevotella* ↑	p < 0.05
		9 dCCA cases	*Streptococcus*, *Prevotella*, *Actinomyces* ↑	p < 0.05
Chen B et al. (31)	2019	8 dCCA patients’ biliary microbiota	*Gemmatimonadetes*, *Nitrospirae*, and *Planctomycetes* phyla ↑ *Chloroflex* ↓	p < 0.05
Chng KR et al. (32)	2016	60 BTCs cases	Predominant inhabitant of BTCs tissue: *Dietzia*,*Pseudomonas* Opisthorchis viverrini infection: *Bifidobacteriaceae* ↑	p < 0.05
F. Avilés-Jiménez et al. (35)	2016	100 CCA cases	Fusobacterium, *Prevotella* ↑ *Rothia* ↓	p < 0.05
Uguz A et al. (36)	2025	Pancreas of 10 dCCA/AC patients	*Prevotella*, *Streptococcus*, and *Fusobacterium* are the oral microbial genera most frequently found to be enriched in pancreatic tissue.	p < 0.05
		Saliva of 10 dCCA/AC patients	Oral microbial diversity was lower in the cancer group. *Provetella*, *Rothia*, *Veillonella*, *Acytinomyces*, *Porphyromonas*, *Fusobacterium* ↑ *Streptococcus*, *Neisseria* ↓	p < 0.05
B.-C. Rao et al. (41)	2022	oral cases of 272 BTC patients	*Firmicutes*, *Fusobacteriota*, *Spirochaetota*, *Synergistota* ↑ *Streptococcus*, *Veillonella*, *Haemophilus* ↑	p < 0.05
Oh S et al. (42)	2025	oral cases of 14 BTC patients and 14 HC	No significant differences between BTC and healthy controls.	P=0.1

This table summarized major clinical studies reporting changes in oral bacterial communities among BTCs patients. It highlights whether specific genera showed increased (↑) or decreased (↓) abundance and provides corresponding statistical measures. BTC, biliary tract cancer; CCA, cholangiocarcinoma; pCCA, perihilar cholangiocarcinoma; dCCA, distal cholangiocarcinoma; AC, ampullary cancer; HC, healthy controls; HR, hazard ratio; CI, confidence interval; p, significance level (p<0.05 indicates statistical significance).

## 4 The oral microbiota and risk factors for BTCs

**Figure 2:**
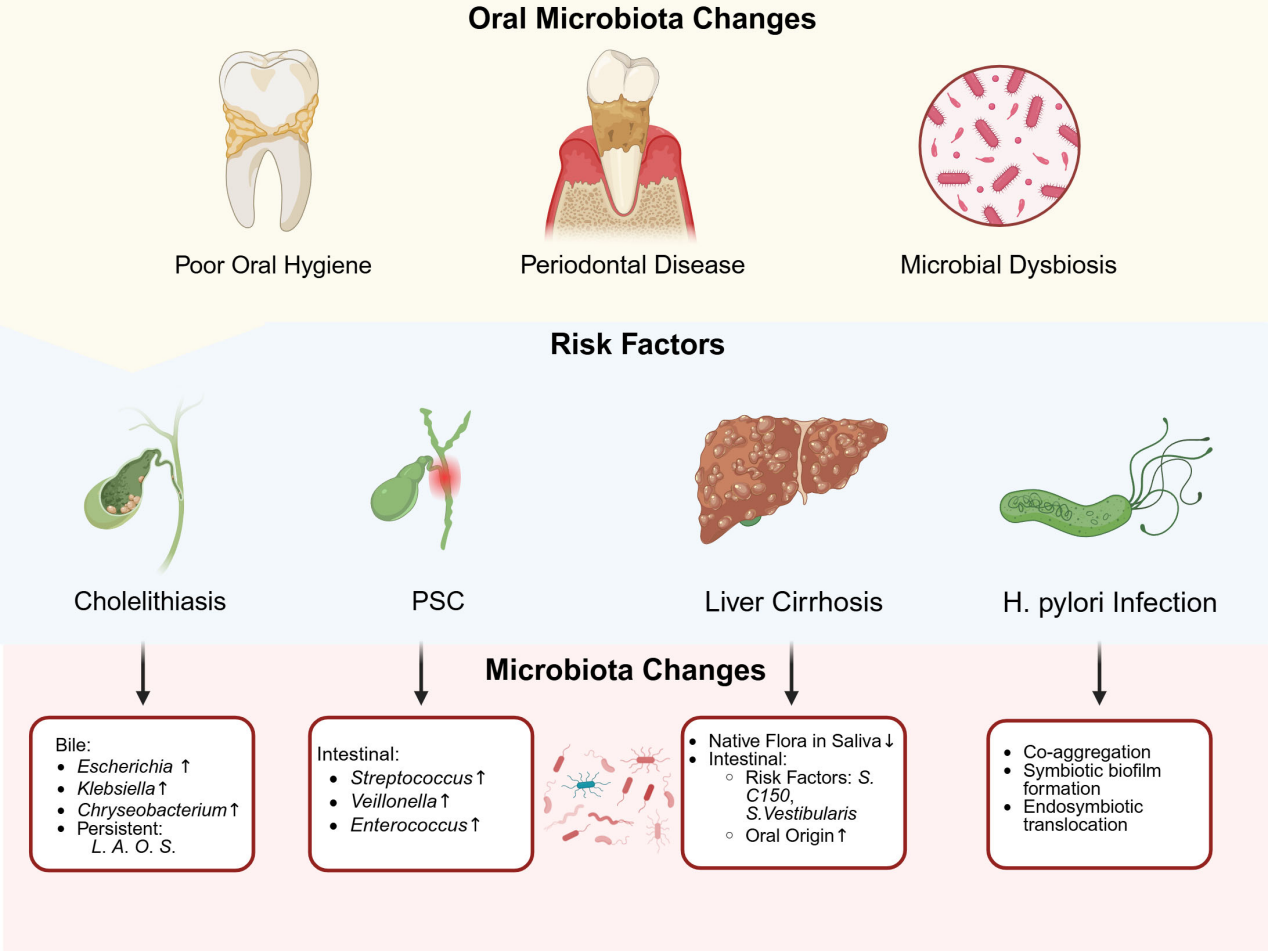
The association between oral microbiota and risk factors in biliary tract cancer. Four established risk factors-gallstones, PSC, liver cirrhosis and Helicobacter pylori infection-were each linked to distinct shifts in oral microbial communities. Gallstones and poor oral health correlate with higher levels of oral bacteria such as Escherichia, Klebsiella and Chryseobacterium in biliary samples, while there was a persistence of *Lachnoanerobaculum* (*L*), *Atopobium* (*A*), *Oribacterium* (*O*) and *Stomatobaculum* (*S*). PSC patients exhibit increased *Streptococcus salivarius*, V*eillonella parvula* and *Enterococcus* in intestinal microbiota. Cirrhosis−related gum disease showed loss of native flora in saliva (e.g., *Streptococcus*) alongside a rise of risk factors (e.g., *S. C150*, S*. Vestibularis*) and oral oringin microbiota in the gut. *H. pylori* infection reshaped oral communities by promoting co-aggregation and the formation of biofilms by species such as *Gemella* and *Holdemanella*, as well as endosymbiotic translocation. These microbial changes may contribute to cancer development through direct colonization, immune modulation and chronic inflammation. Created in https://BioRender.com.

BTCs encompasses two main subtypes: bile duct cancer and gallbladder cancer. Established risk factors for bile duct cancer included primary sclerosing cholangitis (PSC), Caroli’s disease, intrahepatic bile duct stones, and liver fluke infection. Primary risk factors for gallbladder cancer included cholelithiasis and primary sclerosing cholangitis, among others (5). (See **Figure 2** for microbial alterations associated with established BTC risk factors).

### 4.1 Cholelithiasis

Cholelithiasis can occur at any location within the biliary system, encompassing the gallbladder and both intrahepatic and extrahepatic bile ducts (47). Current evidence demonstrated a positive correlation between cholelithiasis and increased risk of biliary tract and gallbladder carcinoma, with notably 70-90% of gallbladder cancer patients presenting with antecedent gallstone disease (48).

Multiple investigations have elucidated the role of microorganisms in cholelithiasis pathogenesis (49). As previously established, oral microbiota maintained intimate associations with oral hygiene status. A population-based survey in the United States demonstrated a significant positive correlation between poor oral hygiene and ultrasonographically confirmed cholelithiasis, further supporting the connection between oral microbiota and gallstone disease (50). Using whole-metagenome shotgun (WMS) and 16S sequencing, Shen et al. identified 25 oral/respiratory tract-derived microorganisms among 54 microbial species in bile samples from patients with common bile duct stones. Notably, these oral-derived bacterial species exceeded the quantity of intestinal-derived species. Furthermore, among 13 newly discovered species in bile, 8 belonged to oral microbial groups, suggesting a substantial association between oral microbiota and cholelithiasis (51). Additional studies using 16S sequencing revealed elevated levels of *Escherichia* and *Klebsiella* in bile samples from cholelithiasis patients, with *Chryseobacterium* also being detected in the bile (52). Moreover, oral microbiota-associated genera including *Lachnoanerobaculum*, *Atopobium*, *Oribacterium*, and *Stomatobaculum* demonstrated persistent presence in the bile of cholelithiasis patients, with their abundance increasing during disease onset and progression (53).

### 4.2 Primary sclerosing cholangitis

PSC represents a chronic inflammatory liver condition characterized by progressive scarring of bile ducts and intrahepatic biliary system, potentially predisposing to BTCs development (54).

Multiple studies have confirmed increased abundances of *Streptococcus*, *Veillonella*, and *Enterococcus* genera in the intestinal microbiota of PSC patients (55–57). Analysis of saliva and fecal samples from PSC patients revealed significant enrichment of eight bacterial species, including *Streptococcus salivarius*, *Veillonella parvula*, *Actinomyces*, and *Bifidobacterium*, suggesting potential oral microbiota translocation and colonization as crucial factors in PSC pathogenesis (57).

### 4.3 Liver cirrhosis

Evidence and mechanisms supporting liver cirrhosis as a premalignant condition for BTCs have been established (58).

As previously noted, periodontal status is closely associated with the oral microbiome (25). A direct causal relationship existed between liver cirrhosis and periodontitis (59). The study involving 164 non-alcoholic fatty liver disease patients demonstrated that those with *P. gingivalis* infection in saliva and deeper periodontal pockets exhibited higher liver stiffness values compared to patients without periodontal disease. Patients with increased liver stiffness showed elevated serum antibody titers against *P. gingivalis* strains FDC381 and SU63. Logistic regression analysis confirmed the correlation between periodontal disease and liver stiffness (60). The subgingival microbiota in cirrhosis-associated periodontitis consisted of a distinct bacterial community that was typically unrelated to conventional periodontitis, likely resulting from dysbiosis due to compromised immune function (61). The oral cavity represented a significant source of inflammation in liver cirrhosis. Periodontal treatment can improve endotoxemia, salivary inflammation, and systemic inflammation in cirrhotic patients, while also regulating dysbiosis in salivary and fecal microbial communities (62).

Regarding the characteristic changes in salivary microbiota of patients with liver cirrhosis, there is a decreased relative abundance of indigenous bacteria (such as *Streptococcus*), while potentially pathogenic families (*Enterobacteriaceae* and *Enterococcaceae*) show significantly increased relative abundance. These findings suggested that oral dysbiosis could be associated with the progression of liver disease (63). In oropharyngeal swabs from cirrhotic patients with pneumonia, compared to those without pneumonia, there were increased populations of *Bacteroides*, *Neisseria*, and *Actinomyces*, while the *Streptococcus* population was decreased (64).

Due to the persistent presence and high abundance of lantibiotics genes in the gut microbiome of cirrhotic patients, oral *Streptococcus C150* and *Streptococcus vestibularis*, which encode lantibiotics before their translocation to the intestine, are potential risk factors for liver cirrhosis (65).

In a quantitative metagenomic analysis of gut microbiome from 98 cirrhotic patients and 83 healthy controls, there were 66 clusters representing homologous bacterial species that differed between the cirrhotic and healthy groups. Among the 28 bacterial species enriched in cirrhotic patients, the majority originated from the oral cavity (66). Japanese researchers have demonstrated that patients with liver fibrosis show increased relative abundance of *Clostridium* strains and decreased relative abundance of *Faecalibacterium* in their gut microbiota (67).

### 4.4 Helicobacter pylori

Specific *H. pylori* strains clearly associate with increased BTCs risk (35, 68).

The oral microbiota, being upstream of the stomach, serves as a primary source of gastric microbiota. Research showed that *H. pylori* infection was not only associated with the degree of coexistence between oral and gastric mucosal microbiota, but *H. pylori* infection itself can also influence the composition of oral bacterial communities through altering local pH or competitive colonization (69, 70). The oral microbiota influenced the transmission and colonization of *H. pylori* through co-aggregation, symbiotic biofilm formation, and endosymbiotic translocation colonization (71).

Interestingly, recent research has shown that abnormal abundances of specific oral bacteria—such as *Gemella* and *Holdemanella*—significantly affect the risk of gastric cancer (43). These species were not only closely linked to gastric cancer and may, through their complex interactions with *H.pylori*, alter the bacterium’s growth and ability to cause disease. Consequently, this could influence the risk of BTCs. Such dysbiosis may impair both oral and gastric mucosal barriers. It can also modulate *H. pylori*’s pathogenicity through bacterial coaggregation, co−formation of biofilms, and internal translocation. These processes could contribute to BTCs initiation and progression. Future research should clarify how oral microbiota and *H. pylori* interact and determine how these microbial interactions influence BTC risk and disease course.

## 5 Conclusions and future directions

Accumulating evidence indicates that the oral microbiota can influence biliary tract carcinogenesis via three principal mechanisms: direct bacterial colonization of biliary tissues, modulation of the local immune microenvironment, and microbial metabolic interactions (21–24). Opportunistic oral bacteria such as *Porphyromonas gingivalis* are capable of ectopically colonizing the bile ducts and triggering chronic biliary inflammation (e.g., through TLR4/NF-κB activation), which in turn promotes abnormal epithelial proliferation and DNA damage. Additionally, bacterial products like OMVs can dampen anti-tumor immune responses for instance, by upregulating the PD-1/PD-L1 immune checkpoint, and can act in concert with host pro-inflammatory cytokines to create a tumor-promoting immunosuppressive niche Under gut-liver axis regulation, *Veillonella*-derived LPS and secondary bile acids cooperatively drived biliary cell metabolic reprogramming through oxidative stress and FXR receptor signaling. These mechanistic links are supported by clinical observations: epidemiological studies have correlated poor oral health (e.g., periodontitis) with elevated risk of BTC, and oral bacterial taxa such as *Fusobacterium* and *Prevotella* have been detected within bile and tumor tissue of BTCs patients.

Contemporary research substantiates a significant correlation between oral microbiota dysbiosis and BTCs risk, with periodontal disease history and poor oral health linked to 73% (HR=1.73) and 32% (HR=1.32) increased risk, respectively (26, 27). Molecular biological evidence has revealed enrichment of oral-origin bacteria genera in bile and biliary tissues of BTCs patients, including *Fusobacterium*, *Prevotella*, and *Streptococcus* (30, 35). Opportunistic pathogens such as *Deinococcus* and *Pseudomonas* may contribute to carcinogenesis through oral-biliary ectopic colonization. Further mechanistic studies indicate that oral bacterial metabolites (such as *P. gingivalis* LPS) can induce chronic inflammation by activating the TLR4/NF-κB pathway, working synergistically with pathways like STAT3 to drive abnormal cell proliferation (39, 41). According to gut-liver axis theory, BTCs patients show intestinal dysbiosis. This condition, marked by increased *Veillonella* and *Enterobacteriaceae*, may promote tumors through AMPK/mTOR-mediated metabolic reprogramming (38). Given the significantly increased oral microbial α-diversity and abnormally elevated abundance of oral dominant bacteria (*Firmicutes*, *Streptococcus)* in gallbladder cancer patients, dynamic changes in oral microbiota showed promise as biomarkers for BTCs development.

Overall, we proposed a novel mechanistic hypothesis in section 3 to explain the variations in oral microbiota diversity observed across different types of BTCs. Dysbiosis of the oral microbiota associated with biliary cancer may have shown a “biphasic pattern”. Specifically, this biphasic pattern refers to the observation that, although both iCCA and dCCA are subtypes of BTCs, oral microbial diversity is higher in iCCA patients than in healthy controls, whereas it is lower in dCCA patients (36, 42). In some cases (e.g., iCCA), weakened immune surveillance and prolonged low-grade inflammation supported the coexistence and proliferation of multiple opportunistic pathogens in the oral cavity, leading to increased microbial diversity. This enriched microbial community continuously released bioactive molecules, such as LPS and metabolic byproducts, which affected liver immune responses. These changes contributed to the development of a chronic inflammatory microenvironment that promoted malignant transformation of biliary epithelial cells (44). In contrast, in other cases (e.g., dCCA), repeated acute inflammation and medical interventions led to colonization by only a few dominant pathogenic species, resulting in reduced diversity (45, 46). Though these dominant microbes had stronger pro-inflammatory and carcinogenic potential. Both two types of dysbiosis eventually contributed to cholangiocarcinogenesis through both direct and indirect pathways mentioned above.

Despite significant progress, current researches exhibits several limitations. The predominance of cross-sectional study designs precluded establishment of clear temporal relationships between oral dysbiosis and BTCs development. Limited sample sizes in some studies (n<50) constrain the identification of specific bacterial signatures (30, 36, 43). Analysis of specific metabolic regulatory networks remains incomplete, particularly regarding the quantification of dynamic concentrations and targets of oral bacterial metabolites in the biliary microenvironment (41). Additionally, spatial heterogeneity of microbiota across gallbladder anatomical subsites requires further investigation.

Future research should validate causality through multicenter longitudinal cohort studies and humanized mouse models. These studies could lead to novel therapeutic approaches targeting metabolic pathways and microbiota. One potential strategy is to leverage machine learning on microbiota data to enable early cancer prediction and diagnosis. Large-scale analyses of oral and gut microbiota from gastrointestinal cancer patients have already enabled machine learning models to distinguish cancer cases with high accuracy (AUC>0.8) (72, 73). These findings encourage the development of AI-driven oral microbiome diagnostics for GI and hepatobiliary cancers as a complement to or even replacement for more invasive screening methods. And multi-omics AI models are being explored: an integrative graph convolutional network that combined microbiome features with exposome data achieved about 90% accuracy (AUROC ~0.89) in detecting pancreatic cancer (74). Larger, long−term studies and validations across diverse populations are needed to bring these AI−powered microbiome models into routine cancer screening. Saccharomyces cerevisiae has been validated as a novel microbial platform for delivering agents to gastrointestinal tumors (75). Future work could engineer strains to express BTCs-specific antigens. This strategy may overcome the poor mucosal penetration and high systemic toxicity of conventional therapies. Additionally, integrating single-cell and spatial omics with metabolic tracing and intelligent delivery systems could accelerate the discovery of tumor–microbe interaction mechanisms. It could also enable microbiome-based early screening and targeted interventions, offering new translational avenues for BTCs that have poor prognosis and lack reliable biomarkers.
